# Prediction and Characterization of CYP3A4-mediated Metabolisms of Azole Fungicides: an Application of the Fused-grid Template* system

**DOI:** 10.14252/foodsafetyfscj.D-20-00010

**Published:** 2020-06-26

**Authors:** Yasushi Yamazoe, Takashi Yamada, Kiyoshi Nagata

**Affiliations:** 1 Division of Drug Metabolism and Molecular Toxicology, Graduate School of Pharmaceutical Sciences, Tohoku University, 6-3 Aramaki-Aoba, Aoba-ku, Sendai 980-8578, Japan; 2 Division of Risk Assessment, National Institute of Health Sciences, Tonomachi 3-25-26, Kawasaki-ku, Kawasaki 210-9501, Japan; 3 Department of Environmental Health Science, Faculty of Pharmaceutical Sciences, School of Pharmaceutical Sciences, Tohoku Medical and Pharmaceutical University, 4-4-1 Komatsushima, Aoba-ku, Sendai 981-8558, Japan

**Keywords:** Key word CYP3A4 inhibition and metabolism, triazole, imidazole, fungicide

## Abstract

Human CYP3A4 is involved in metabolisms of diverse hydrophobic chemicals. Using the data of therapeutic azole fungicides known to interact with CYP3A4, applicability of CYP3A4 Template system was first confirmed to reconstitute faithfully the interaction on Template. More than twenty numbers of pesticide azoles were then applied to the Template system. All the azole stereo-isomers applied, except for talarozole, interacted through nitrogen atoms of triazole or imidazole parts and sat stably for inhibitions through fulfilling three-essential interactions. For their CYP3A4-mediated oxidations, clear distinctions were suggested among the enantiomers and diastereomers of azole pesticides on Templates. Thus, the stereoisomers would have their-own regio- and stereo-selective profiles of the metabolisms. A combined metabolic profile of each azole obtained with CYP3A4 Template system, however, resembled with the reported profile of the *in vivo* metabolism in rats. These results suggest the major roles of CYP3A forms on the metabolisms of most of azole pesticides in both rats and humans. Free triazole is a metabolite of azole fungicides having a methylene-spacer between triazole and the rest of the main structures in experimental animals and humans. During the simulation experiments, a placement for the oxidation of a methylene spacer between the triazole and main carbon-skeleton was found to be available throughout the azole fungicides tested on Template. The occurrence of this reaction to lead to triazole-release is thus discussed in relation to the possible involvement of CYP3A forms.

## 1. Introduction

Imidazole and triazole fungicides are used as clinical medicines and pesticides. These chemicals influence sterol biosyntheses^1^^,^^2^^,^^3^^)^ and also retinoic acid metabolisms^4^^,^^5^^)^, which may be associated with the adverse outcomes^6^^,^^7^^,^^8^^)^. These fungicides showed broadened biological half-lives in experimental animals and humans. The metabolic fates in humans of fungicides used clinically are investigated thoroughly at the level of the enzyme-form involved and also properties of the metabolites, which may be necessary to understand the individual differences in their pharmacokinetics. The metabolic fates in human of pesticides, however, remain mostly undefined, although the metabolisms in rodents are available.

In the toxicological evaluation of pesticides, data obtained with experimental animal species are used for the extrapolation to assess the safety in humans. Both qualitative and quantitative similarities of pesticide metabolisms among the species are one of the major criteria for the extrapolation. To cope with the situation, various systems have been introduced to predict human drug metabolisms.

Cytochrome P450 (CYP) is involved in the metabolism of diverse hydrophobic chemicals. Uses of crystalized CYP data afforded information on the active sites of various human-CYP forms, but the rigid prediction of ligand interactions are still not easy tasks at present.

We developed prediction systems for ligand interactions of seven human CYP-forms using grid-based Templates^9^^,^^10^^,^^11^^,^^12^^,^^13^^,^^14^^,^^15^^,^^16^^)^. These Templates are constructed with assemblies of ligand interactions and constituted as flat fused-hexagonal grids. The shapes of Templates are defined through reciprocal comparisons of simulation data with experimental results of recombinant CYP-reaction systems. In these Template systems, ligand interactions are shown as placements on specific Template of these CYP forms after considerations of regional interactions. As the results, Template systems offered the placements to predict sites of metabolisms regio- and stereo-selectively with more than 99% of accuracies with CYP1A1 (>350 reactions)^16^^)^, CYP1A2 (>450 reactions)^17^^)^ and CYP3A4 (>1,080 reactions)^18^^)^. The failures (inconsistency) are due to the secondary phenomena like NIH-shift and nonenzymatic cyclization in these reactions.

CYP3A4 is a major CYP form expressed in livers and small intestines of humans, and mediates oxidations and reductions of chemicals having diverse structures. Inhibition of CYP3A4 with hydrophobic ligands including azole fungicides is often associated with adverse outcomes in chemotherapeutics. Therefore, several triazole and imidazole pesticides are applied on the Template in the present study to verify the possible role of human CYP3A4 on azole pesticide metabolisms.

## 2. Materials and Methods

Experimental information on the substrate specificities and metabolites on CYP3A4 substrates was obtained from published literatures. The data on recombinant human CYP3A4 systems were used preferably because these data indicate the contribution of CYP3A4 distinctly. Chem3D (version 5 for Mac OS, CambridgeSoft, Cambridge, MA) and ChemBio3D (Version 12 for Windows, CambridgeSoft) and ChemBioDraw (PerkinElmer) were used to constructtwo- (2D) and three-dimensional (3D) structures of the substrates and to overlay compounds.

Substrates of CYP3A4, except for PAHs, take various conformations due to their flexibility. Prior to the Template application, chemicals are taken in their flattened form(s). The flattened or extended shapes of the 3D structures were tried to sit on Template and then modified their conformations to fit within Template, in consideration of the bond-energy barrier using MM2 function of Chem3D and also specific interaction at distinct regions of Template^14^^,^^15^^)^. Carbon, oxygen, nitrogen, sulfur and halogen atoms of 3D ligand structures are indicated with gray, red, blue, yellow and green symbols, respectively. The hydrogen atoms of the substrates were not considered for the placement.

Template consists of hexagonal grids and sticks. The sitting of substrate atoms at each corner of the hexagonal-grids (termed Rings) was evaluated as occupancy. The placement of substrates in text is expressed in a hyphen-linked form, such as Rings A-B-C, to trace the occupancy of chemical molecules on Template. The sizes and locations of Bay-1 residue, Bay-2 residue and Front-residue, and of Cavity-1 residue, Cavity-2 residue and Groove were defined in the second and third studies of CYP3A4 Template, respectively^14^^,^^15^^)^. Template is updated with Positions 5’ and 9’ and half-Ring regions Q’ and W’ in the fourth study^18^^)^.

**Fig. 1.**
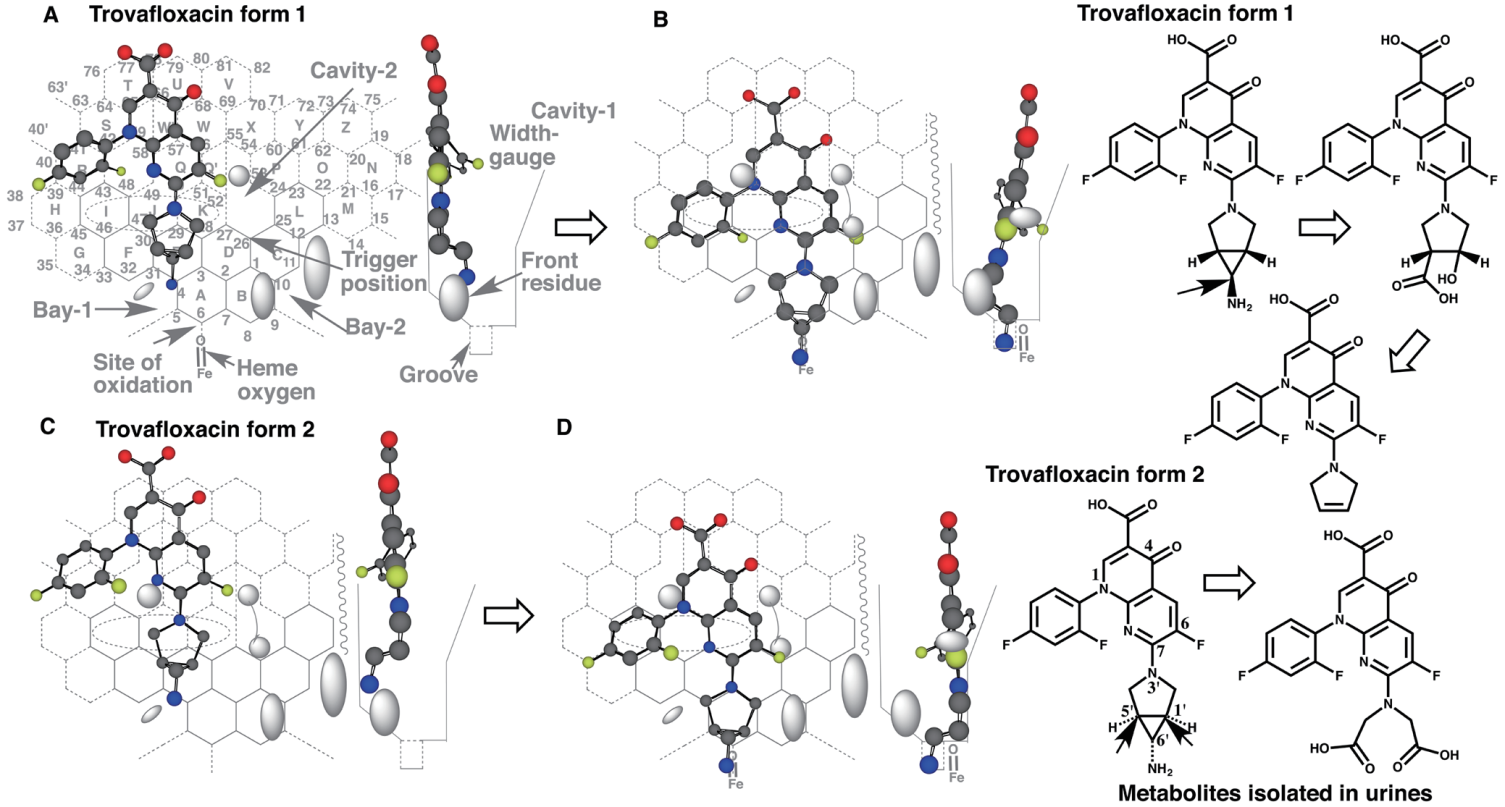
Template of CYP3A4 A flat Template of CYP3A4 is shown with Ring names and Position numbers (A). Core and expanded areas are drawn as normal and dotted lines. There are four specific regions on Template (Bay-1, Bay-2, Cavity-1 and Cavity-2). Bay-1 is surrounded by Positions 5, 4, 31 and 32. Bay-2 is located near Positions 11, 12 and 13. Cavity-1, surrounded by Positions 42, 43, 48, 49, 58, 66, 65 and 59, is devoid of ligand occupancy. Cavity-2, surrounded by Positions 24, 25, 26, 27, 52, 55, 54 and 53, is also not occupied with ligands. Site of oxidation (Position 6/6’) and Trigger-site (Position 26) are indicated with dark arrows. Width-gauge was shown with connected lines in the right side as 90°-rotated figure. A ligand-sitting ditch termed Groove is located at the middle of the bottom of Width-gauge. The round symbol on Width-gauge is illustrated as Front residue. Symbol sizes of Cavity-1, Cavity-2 and Front residues are rough estimates derived from simulation results of CYP3A4 ligands on Template (A and B). A gray dotted circle indicates a region of IJK-interaction (A and B). Ligands are expected to enter from left-side of Template, and then Cavity-1 and Cavity-2 residues appear on Template plane. Trovafloxacin is able to enter into Template in two distinct ways shown as form 1 and form 2.

An example of Template application is shown using a presumed CYP3A4 substrate, trovafloxacin^19^^)^ (Fig. 1). Trovafloxacin, released on the market in 1998, was withdrawn from the market due to rare, but severe, acute liver failure. The mechanism underlying trovafloxacin-induced hepatotoxicity still remains to be characterized^20^^)^

Two distinct conformers of trovafloxacin enter in CYP3A4 Template from the left-side (Rings G, H, R, S and T) and move to the right-side until contacting Cavity-2 region (Figs. 1A and C). Both molecules differ in their orientations of the pyrrolidine-cyclopropyl ring part due to the rotatable C-N bonding to the quinoline moiety. No conformation changes are assumed during the migration of ligands, and thus ligands migrate as their conformations the same as observed at Site of oxidation in Template. In addition, Cavity-1 residue is assumed to appear on Template after a ligand passage and thus does not interfere the ligand migration into Template.

The contact of trovafloxacin molecules at Rings I, J and/or K with Template facilitates the shift of the ligand molecules to facial-side of Width-gauge. The ligand molecule slides down to Rings A and B after passing through the gate consisted of Bay-1 and Cavity-2 residues. The amino groups on cyclopropyl ring stick in Groove. The carbon atom connecting primary amino group (Fig. 1B) and carbon atoms at the bridge part of cyclopropane and pyrrolidine rings (Fig. 1D) thus face to contact with heme-oxygen.

Ligands interacts at Rings I, J and/or K (IJK-Interaction), at Position 6 (Site of oxidation) and at Position 26 (Trigger position) on Template for their functional contributions (oxidation/reduction/inhibition). All the three distinct interactions are fulfilled in single molecule in uni-molecule binding.

Two molecules are simultaneously accommodated as pro-metabolized and trigger molecules in bi-molecule binding. Pro-metabolized molecule needs to fulfill IJK-Interaction and contact at Site of oxidation in bi-molecule binding. Both pro-metabolized and trigger molecules sit within Width-gauge, which is a guide shown as 90°-rotated view from Ring N side (Fig. 1 Right sides).

Both placements of trovafloxacin (Figs. 1B and D) suggest the predominant *C*-oxidation rather than the *N*-oxidation, in spite of the terminal localization of the primary amino group. The result was consistent with the profile of the oxidized metabolites *in vivo* in rats (Fig. 1, 2D structures of metabolites)^19^^)^, although the conjugations are reported as the main route of trovafloxacin excretion in humans^21^^)^. Thus, the regio- and stereo-selectivity of the metabolite formation is judged from modes of ligand sittings at Site of oxidation and of heme-oxygen access on CYP3A4 Template.

## 3. Results

To understand the modes of interaction of triazole ligands with CYP3A4, fluconazole and voriconazole are chosen at first for Template application because of the known metabolic-fates in humans. Azole chemicals interact with CYP3A4 as both inhibitors and substrates, and higher affinities are mostly detected on their inhibitions rather than on their oxidations. Thus, placements for the inhibition are described at first and then followed the placements for oxidations in Results.

### 3.1 Placements of Fluconazole and Voriconazole

Fluconazole inhibits CYP51 catalyzing ergosterol 14α-demethylation^22^^)^ and CYP26 involved in the maintenance of retinoic acids^23^^)^. This chemical also inhibits drug-metabolizing CYP forms such as CYP2C9 and CYP3A4^24^^)^. Majority of fluconazole was excreted as unchanged in urine after the oral administration. Less than 10% of the dose was detected as the *N*-oxide in humans^25^^)^. Triazole was isolated in urines of mice and dogs^26^^)^.

**Fig. 2.**
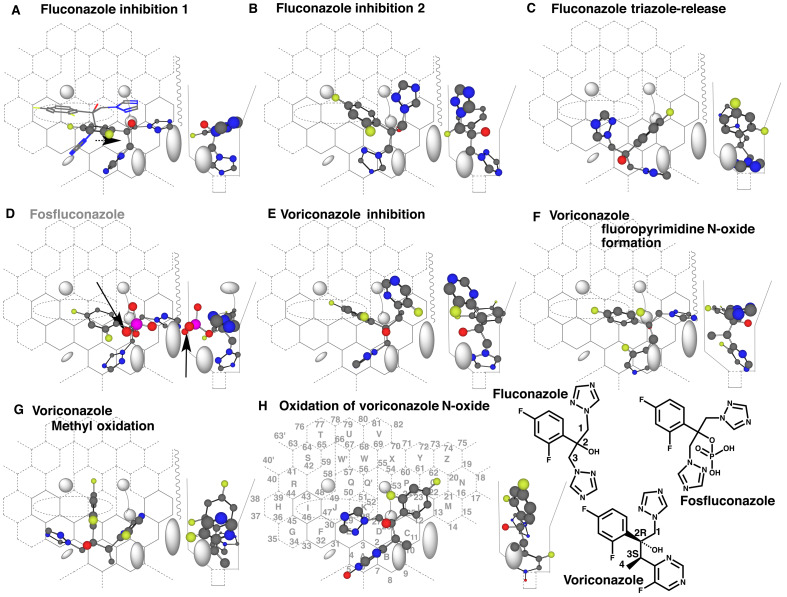
Placements of fluconazole, fosfluconazole and voriconazole Placements of fluconazole for inhibition (A and B) and triazole-release (C) are shown as cylindrical-shape 3D structures on CYP3A4 Template with the 90°-rotated structures on width-gauge. Passage of fluconazole molecule through a gate consisted of Bay-1 and (upper)-Cavity-2 residues is show as stick-shape structure in A. A possible sitting of fosfluconazole is shown in D. The phosphate ester part exceeded the facial-side border as indicated with arrows. Placements of voriconazole for inhibition (E), *N*-oxide formation (F) and methyl oxidation (G) are shown on Template. A placement of voriconazole *N*-oxide for the fluoropyrimidine-ring oxidation is shown in H. Oxygen atom of the *N*-oxide sat in Groove, and thus the molecule was immobilized. Non-functional placement is indicated in gray color of structure name. 2D-Structures of fluconazole, fosfluconazole and voriconazole are shown with parts of position numbers in the bottom.

A placement for fluconazole inhibition of CYP3A4 was available at Rings A-B(C-L)-D-E-J (Fig. 2A, cylindrical-shape). This conformation was allowed to pass a gate between Bay-1 and Cavity-2 residues (Fig. 2A, stick-shape), and satisfied all the three interactions at Rings I, J, and/or K region, Site of oxidation and trigger site after the slide-down to Rings A and B. Flipping of the triazole ring at Ring A might offer a chance of *C*-oxidation of triazole ring, but the Futile-sitting (rotation)^18^^)^ would negate the *C*-oxidation.

On CYP3A4 Template system, Bay-1 and (upper)-Cavity-2 residues form a gate to restrict ligand entrance into Template. (Upper)-Cavity-2 residue, however, allows thin-shape ligands to pass from simulation experiments of macro-ring ligands such as α-zeranol and SB1317^27^^)^. These thin-shape ligands were expected to move to the right-side region after the facile-side shift through IJK-Interaction. These data suggested an existence of a thin open-space at the facial-side of (upper)-Cavity-2 residue. Triazole part of fluconazole might move to the right-side region of Template through the pass at (upper)-Cavity-2 residue (Fig. 2B).

A placement for the possible oxidation of 2,4-difluorophenyl part of fluconazole was constructed at Rings A-D(C-L)-K-J plus Position 6’, which fulfilled the requirement of the three interactions (Data not shown). The fluconazole molecule was, however, not expected to pass readily the gate between Bay-1 and (upper)-Cavity-2 residues, suggesting the scare chance of CYP3A4-mediated oxidation of the 2,4-difluorophenyl part.

Free triazole is isolated as a metabolite in the excreta of animals given various fungicides having methylene spacer between triazole and the rest part including fluconazole. A placement for the triazole release of fluconazole was generated at Rings A(B/D-C)-E(F)-J (Fig. 2C). Introduction of a hydroxyl group or hydrogen-atom abstraction at the methylene part might resulted in the enamine-type intermediate formation, and the subsequent spontaneous hydrolysis of the intermediate would yield triazole and an alcohol metabolite containing the rest part of the molecule.

A phosphate ester of fluconazole, fosfluconazole, shows no clear spectral interaction with recombinant CYP3A4^28^^)^. A placement of fosfluconazole was constructed at Rings A-B-C(L)-D-K for the possible inhibition (Fig. 2D). The phosphate group exceeded the border of facial-side wall, suggesting the difficulty to accommodate fosfluconazole molecule for the interaction at Site of oxidation.

CYP3A4 mediates both the fluoropyrimidine *N*-oxide formation^29^^,^^30^^)^ and methyl oxidation^30^^)^ of a chiral triazole, voriconazole. Fluoropyrimidine ring of the *N*-oxide is further oxidized to the hydroxyl derivative in various species including humans^31^^)^.

A placement for the inhibition of voriconazole was available at Rings A-B-C(L-M)-D-K-J (Fig. 2E). The nitrogen atom of triazole sat close to Position 6 (Site of oxidation) and a chiral center (2R) connecting 2-hydroxyl group served for triggering.

Sitting of voriconazole molecule at Rings A-B-C(L)-D-K-J (Fig. 2F) offered a placement for 1’-*N*-oxide formation of the fluoropyrimidine ring.

Unique opened-book-like or fan-shape placement was generated at Rings A(D-C)-E(F)-K. Methyl group at position 4 sat near Site of oxidation (Fig. 2G). Slight exceeding of ligand molecules into Bay-1 region is allowed, and both Bay-1 residue and Front-residue would support the stable standing of this placement.

Clearance rate of voriconazole *N*-oxide is rather higher than the rate of voriconazole in rat and dog, and the observed sex-related difference of the clearance in rats suggest the role of CYP-mediated metabolism^31^^)^. A placement for the fluoropyrimidine ring-oxidation of the *N*-oxide was available at Rings A-B-C(L)-D-K-J plus position 5’ (Fig. 2H). Sitting of oxygen atom of the N-oxide in Groove at Position 5’ was expected to fasten the fluoropyrimidine ring to facilitate the 6’-oxidation. The simulation result was consistent with experimental observations on the rapid metabolism described above. Triazole was detected *in vivo* in rat and dog, but not in human^31^^)^. A placement for the triazole-release of voriconazole was able to be constructed on CYP3A4 Template (Data not shown) in a manner similar to the fluconazole (Fig. 2C). CYP3A4-mediated triazole-release might not be significant for voriconazole due to the reduced order of the placement usages^14^^,^^18^^)^; the primary order observed experimentally is the *N*-oxide formation, followed by *C*-oxidation of the fluoropyrimidine and fluoropyrimidine *N*-oxide, methyl oxidation and triazole release in the decrease order^31^^)^.

### 3.2 Placements of Myclobutanil, Hexaconazole and Propiconazole

Myclobutanil is used as racemate and oxidized in a stereoselective manner in the body^32^^,^^33^^)^. This fungicide is metabolized extensively in rats and thus the parent chemicals represent a few percent of the total excretion^32^^)^. Both R(-)- and S(+)-myclobutanil inhibit human CYP3A-mediated oxidations, but clear differences are detected on the rates of CYP3A4-mediated R(-)- and S(+)-myclobutanil metabolisms *in vitro* determined as the substrate disappearance^34^^)^.

**Fig. 3.**
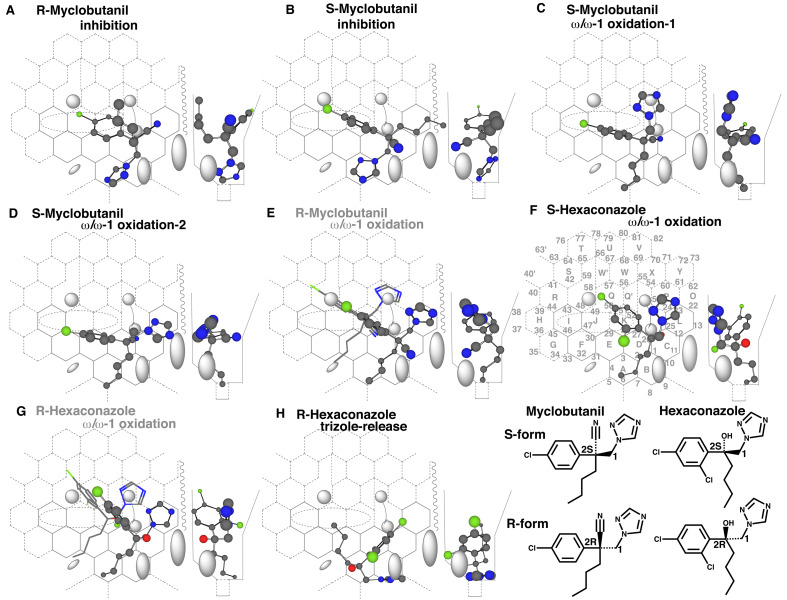
Placements of enantiomers of myclobutanil and hexaconazole Placements for the inhibition of R- (A) and S-myclobutanil (B) enantiomers, for the ω/ω-1-oxidation of the S- (C and D) and R-enantiomers (E) are shown as cylindrical-shapes of 3D-structures. Placements for ω/ω-1-oxidations of S- (F) and R-hexaconazole (G) and for the methylene oxidation of R-hexaconazole (H) are shown as cylindrical-shapes of 3D-structures. Stick-shape structures are also shown to indicate the difficulty to pass a gate of Bay-1/Cavity-2 residues in E and G. Non-functional placements are indicated in gray color of structure name (E and G). 2D-Structures of both azoles are shown with parts of position numbers in the bottom.

Placements for the inhibition of R(-)- and S(+)-myclobutanil were available at Rings A-B-D(C-L/K-Q’)-K-J (Fig. 3A) and at Rings A-D(C-L-M)-K-J (Fig. 3B), respectively. Their triazole parts sat at Site of oxidation to interact with heme, and chlorophenyl parts were located for IJK-Interaction. The acetylene group of R(-)-myclobutanil and the butyl group of S(+)-myclobutanil served for triggering. These results were consistent with the idea of the inhibition of CYP3A4 through triazole interactions for both R(-)- and S(+)-myclobutanil.

Two placements differing the triazole-ring sittings were generated for ω- and ω-1 oxidations of the butyl group of S(+)-myclobutanil at Rings A-D(C)-E-J-I plus facial-side of Cavity-2 or Ring L (Figs. 3C and D). Both the triazole parts located around facial-side walls were expected to pass an open space at (upper)-Cavity-2 residue. A similar placement was constructed for the oxidation of the butyl group of R(-)-myclobutanil at Rings A-D(C-L)-K-J (Fig. 3E), but the molecule was unable to pass the gate of Bay-1/Cavity-2 residues due to the rear-side arrangement of the triazole group. These results were consistent with a report on non-substantial disappearance of the R(-)-isomer in the recombinant-CYP3A4 system^34^^)^. Similar difference is also observed on another enzyme, CYP2C19, for oxidations of myclobutanil^34^^)^. These data suggested the distinct pharmacokinetic profiles of R(-)- and S(+)-myclobutanil in humans. Of course, CYP2C18 oxidizes myclobutanil racemate^35^^)^. Preliminary experiments using Templates of human CYP2C forms suggested CYP2C18-mediated the butyl-chain oxidations of R(-)-myclobutanil (Yamazoe unpublished data). R(-)-myclobutanil might thus undergo the metabolism through a pathway similar to that of S(+)-myclobutanil in humans, although the contribution was low due to the low abundance of CYP2C18 in livers.

Hexaconazole having *n*-butyl side chain is also used as racemate. Clear sex-related difference was known on disappearance rates of the R(-)- and S(+)-hexaconazole in hepatic microsomes of rats^36^^)^.

A placement for the ω/ω-1 oxidation of S(+)-hexaconazole was available at Rings A-D(C-L-P)-K-Q plus a part of Cavity-2 region (Fig. 3F). The triazole part was located around facial-side wall, and thus the molecule was expected to pass the gate of Bay-1/Cavity-2 and did not interfere the descending of Cavity-2 residue. Similar placement of R(-)-hexaconazole was also possible on Template at Rings A-D(C-L)-K-Q. The triazole ring of R(-)-hexaconazole sitting at rear-side prevented from the passage through the gate of Bay-1/Cavity-2 (Fig. 3G). These results suggest the significant contribution of CYP3A forms on S(+)-hexaconazole metabolisms in humans as well as rats. In consistent with this simulation results, the involvement of CYP3A4 is reported on racemic hexaconazole oxidations^37^^)^.

A placement for the methylene bridge oxidation of R(-)-hexaconazole, which would lead to triazole release, was generated at Rings A(B/D-C)-E-J (Fig. 3H). Contact of the butyl side chain was minimal, but attained for IJK-Interaction.

**Fig. 4.**
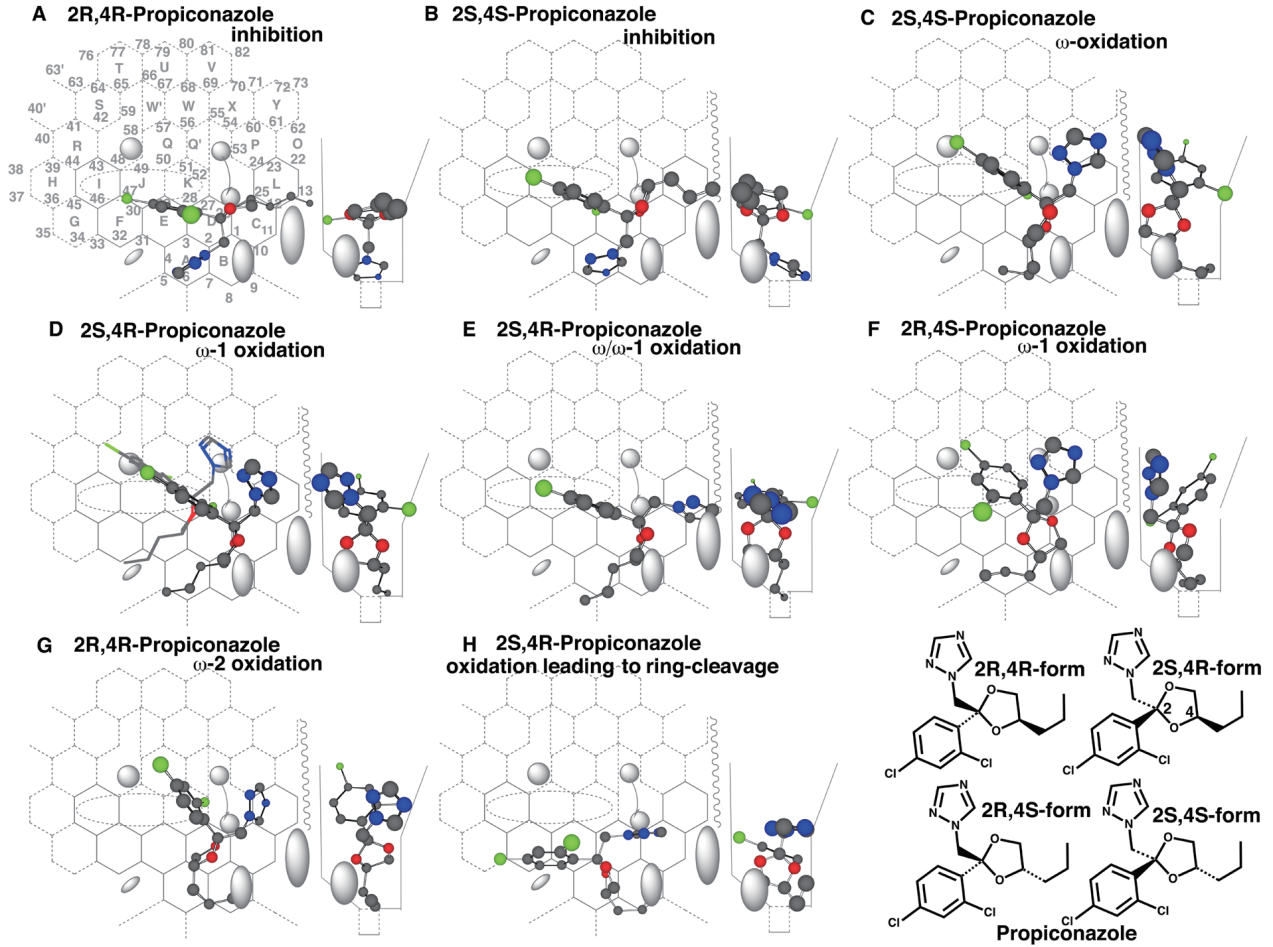
Placements of diastereomers of propiconazole Placements for the inhibition of 2R,4R- (A) and 2S,4S-propiconazole (B), for the side-chain oxidations of 2S,4S- (C), 2S,4R- (D), 2S,4R- (E), 2R,4S- (F) and 2R,4R-diastereomers (G) of propiconazole, and for the oxidation of dioxolane ring of the 2S,4R isomer (H) are shown as cylindrical-shapes of 3D-structures. A stick-shape structure is shown to indicate the escape of triazole-methylene bended to facial side-wall from hitting to Cavity-2 residue (D). 2D-Structures of propiconazole diastereomers are shown with parts of position numbers in the bottom.

Propiconazole containing a dioxolane ring is distributed commercially as a mixture of four stereoisomers. This fungicide was metabolized mainly through oxidations of the *n*-propyl-dioxolane ring parts in rodents^38^^)^. All the 2R,4R-, 2R,4S-, 2S,4R- and 2S,4S-propiconazoles were able to interact through triazole nitrogen atoms to inhibit CYP3A4 on the Template (Figs. 4A and B, only the placements of 2R,4R- and 2S,4S-forms are shown).

A placement for the ω-oxidation of the 2S,4S-form was available at Rings A(B)-D(C-L-P)-K-Q (Fig. 4C). The triazole located at facial-side was allowed to pass (upper)-Cavity-2 residue during the migration into Template. The additional ω-oxidation, possibly also the ω-1 oxidation, was expected from a placement of the 2S,4R-form at Rings A-B-D(C-L)-K-J (Fig. 4D). The triazole ring contacted with facial-side wall and dioxolane ring contacted with Front-residue, which would contribute to the immobilization of the alkyl chain.

Two placements for the ω-1 oxidations of 2S,4R- and 2R,4S-forms were generated at Rings A-B-D(C-L-P)-K-J (Fig. 4E) and at Ring A-B-D(C)-K-Q plus Cavity-2 and Ring P (Fig. 4F). Both the triazole parts were able to pass an open space at facial-side of (upper)-Cavity-2 (Fig. 4E stick-shape) and thus both the placements were judged to be functional on Template. A placement for the ω-2 oxidation of the 2R,4R-form was available at Rings B-A-D(C-L)-K-Q (Fig. 4G). In addition, a placement for an oxidation of the oxolane ring, to lead to the formation of 1-(2,4-dichlorophenyl)-2-(1,2,4-triazol-1-yl)-ethenone, was constructed for the 2S,4R-form at Rings B-A-D(C)-E-F-I (Fig. 4H). Sitting of the propyl part in the aisle behind of Front-residue would support the immobilization of the position 4 of the dioxolane ring. Multiple mono-hydroxylated metabolites, although their structures are not identified, are detected by LC-MS analyses of CYP3A4-mediated oxidations of propiconazole^37^^)^. These results were consistent with the present simulation results.

### 3.3 Other Triazoles Having a Methylene-spacer

**Fig. 5.**
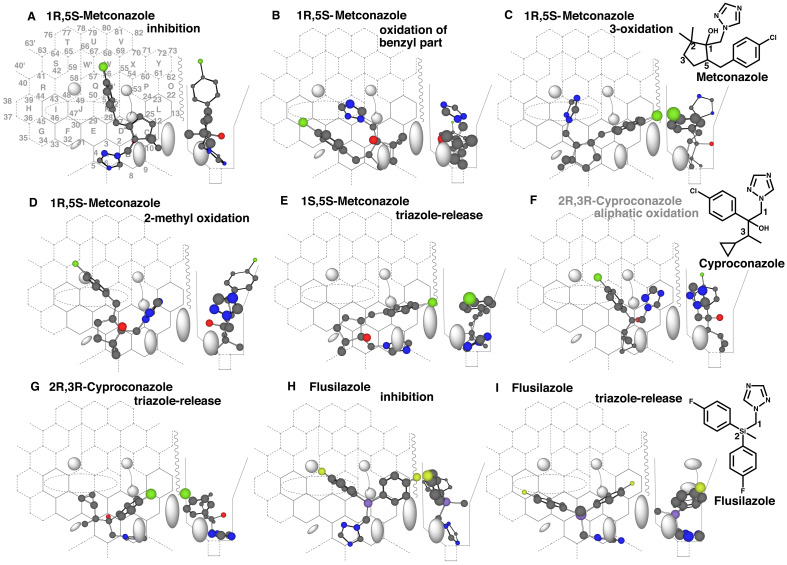
Placements of metconazole and cyproconazole diastereomers and of flusilazole Placements of 1R,5S-metconazole for the inhibition (A), oxidation of the benzyl part (B), 3-oxidation (C), 2-methyl oxidation (D), and of 1S,5S-metconazole for the oxidation of the methylene connecting the imidazole (E) are shown as cylindrical-shapes of 3D-structures. Placements for the aliphatic oxidation of 2R,3R-cyproconazole (F), and for the triazole-release of 2S,3S-cyproconazole (G) are shown on Template. The sitting shown in F was expected to be non-functional due to the lack of trigger occupancy (shown as gray color structure name). Placements of flusilazole for the inhibition (H) and triazole-release (I) are shown as cylindrical-shapes of 3D-structures. 2D-Structures of metconazole, cyproconazole and flusilazole are shown with parts of position numbers in the bottom.

Metconazole also has two chiral centers on the cyclopentane part. This fungicide is oxidized mainly to hydroxylated metabolites of the cyclopentane parts in rats^39^^)^. Inhibitions of CYP3A4 were expected from interactions of their nitrogen atoms of triazole parts. Only a placement of the 1R,5S-form at Rings A-B-C-D-KQ’-W was shown on Template (Fig. 5A).

A placement for the oxidation of the benzyl part of the 1R,5S-form was available at Rings F-E-A-B(C)-D-K (Fig. 5B). The oxidations of positions 3 and 2 of the cyclopentane part of the 1R,5S-form were generated at Rings A(D-C)-E-F plus Position 5’ and above of Bay-2 residue (Fig. 5C) and at Rings A(B-C)-E-J-I plus Position 6’ (Fig. 5D), respectively. In addition, a placement for the methylene oxidation to lead to triazole-release was constructed for the 1S,5S-form (Fig. 5E), but not the 1R,5S-form. These results suggested the dissimilarity of the metabolism among the diastereomers of metconazole and the similarity of the metabolic pathways in rodents and humans.

Cyproconazole having a cyclopropyl part (Fig. 5) is composed of four stereoisomers like propiconazole (Fig. 4). All the isomers (2R,3R-, 2R,3S-, 2S,3R- and 2S,3S-forms) were able to interact with heme of CYP3A4 through nitrogen atoms of their triazole parts (Data not shown) as observed with other triazole fungicides described above.

Placements for the oxidations of the cyclopropyl-ethyl part were constructed on Template, but all the placements were judged to be non-functional substantially due to their insufficient lengths of aliphatic side chains (Fig. 5F, only the placement of the 2R,3R-form is shown). Instead, involvements of CYP2C9 were more likely to occur on oxidations of the cyclopropyl part from Template of CYP2C forms (Yamazoe, unpublished data). Placements for the 1-methylene oxidation to lead to triazole-release were available at Rings B-A(D-C-L)-E-F plus Bay-1 region (Fig. 5G, only a placement for the 2S,3S-form is shown).

A placement of flusilazole for the inhibition was available at Rings A-D(C-L-O)-K-Q (Fig. 5H). The fluorophenyl part at Ring L stood at the facial-side and thus was able to pass the gate between Bay-1 and Cavity-2. No placement for the oxidation of fluorophenyl ring was constructed on Template, but a placement for the oxidation of the methylene bridge was generated at Rings B-A-E(D-C)-F-G (Fig. 5I). The methylene bridge oxidation would result in human CYP3A4-mediated formation of 1,1-difluorophenyl-ethanol and triazole. In consistent with this simulation result, the alcohol is detected as the major metabolite in rats after the oral administration of flusilazole^40^^)^.

### 3.4 Placements of Triadimefon and Other Triazoles Lacking a Triazole-connecting Methylene Bridge

**Fig. 6.**
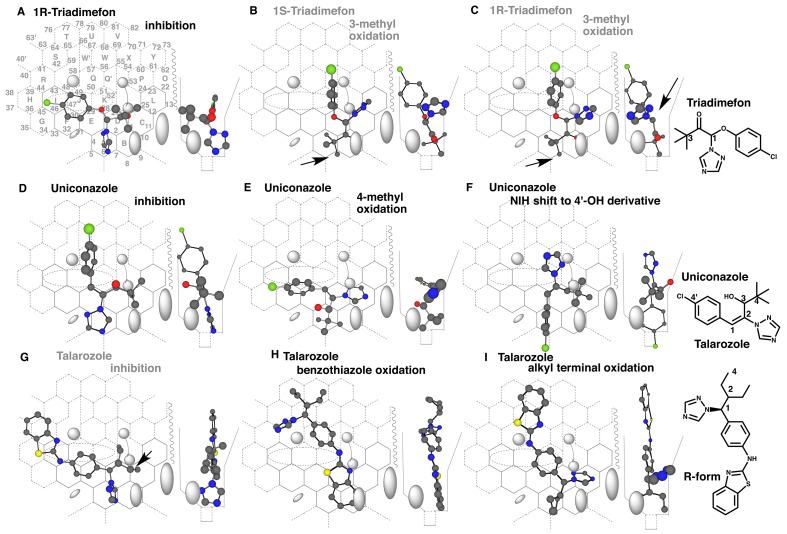
Placements triazoles lacking a triazole-connecting methylene bridge Placements of 1R-triadimefon for the inhibition (A) and of 1S- (B) and 1R-triadimefon (C) for the 3-methyl oxidation are shown as cylindrical-shapes of 3D-structures. Both 1S- and 1R-triadimefon molecules resulted in the overpass of the tert-butyl part after Right-side movement (B and C). Triggering may not be accomplished due to a facial-side sitting of the triazole ring (C). Placements of uniconazole for the inhibition (D), 4-methyl oxidation (E) and oxidation of chlorophenyl ring (F) are shown on Template. The 1,2-double bond takes trans-configuration. Placements of talarozole for the inhibition (G), oxidations of benzothiazole ring (H) and of alkyl terminal (I) are shown as cylindrical-shapes of 3D-structures. Arrows indicate the possible causes of defects. 2D-Structures of triazoles described above are shown with parts of position numbers in the bottom. Non-functional placements are indicated with gray-color structure names.

Some triazole-type fungicides like triadimefon have their structures, in which triazole parts bond directly with their skeletons containing halophenyl parts. Triadimefon is a racemate of the 1R- and 1S-isomers. A placement of 1R-triadimefon for the inhibition was available at Rings A-D(C)-K-J-I (Fig. 6A). Similar placement was also constructed for 1S-triadimefon (Data not shown). Both the 1R- and 1S-isomers were thus expected to interact with their triazole parts to heme of CYP3A4. Although 11β-hydroxysteroid dehydrogenase type 1-mediated reduction of the carbonyl group occurs on triadimefon^41^^)^, methyl oxidation of *tert*-butyl part of triadimefon is observed in the metabolism in rats^42^^)^. A placement for the 3-methyl oxidation of 1S-triadimefon was available at Rings A-B-D(C-L)-K-Q’ (Fig. 6B). The fluorophenyl and triazole parts contacted with facial- and rear-side walls to be fastened. The rear-side sitting of the triazole part was suitable to hold Cavity-2 residue descended for triggering. The *tert*-butyl part was located around Position 7, instead of Position 6. Similar placement for the 3-methyl oxidation of 1R-triadimefon was constructed at Rings A(B)-D(C-L)-K-Q’ (Fig. 6C). The facial-side, but not rear-side, sitting of the triazole part was unlikely to support the triggering. These results suggested scare functional contribution of human CYP3A4 on triadimefon oxidation, which was consistent with the experimental data using recombinant CYP3A4^35^^)^. Both Rat CYP2C6 and human CYP2C19 are reported to mediate the oxidation of triadimefon^35^^)^.

Uniconazole has a double bond which takes *trans*-configuration between chlorophenyl and triazole rings. This chemical is oxidized mainly into the hydroxymethyl derivative of *tert*-butyl part and then the carboxylic acid in rats^43^^)^. A placement for the inhibition of uniconazole was available at Rings A-E(D-C-L)-J-Q-W’ (Fig. 6D). The *tert*-butyl part immobilized through contacts with Front-residue and rear-side wall would serve for triggering in the placement. Formation of the hydroxymethyl derivative was expected from the placement at Rings A-D(C)-E-J-F (Fig. 6E). A metabolite derived from the oxidation of the chlorophenyl part through NIH-shift of the arene oxide is detected *in vivo* in rats^43^^)^. A placement for the 3’,4’-arene oxide formation was generated at Rings A-D(C)-K-Q’ plus Position 6’ (Fig. 6F), suggesting the involvement of CYP3A4.

Talarozole, which is effective in treatments of psoriasis and acne, inhibits selectively retinoic acid-metabolizing enzyme (CYP26A1) rather than CYP51 (sterol 14-demethylase) and CYP19 (aromatase)^44^^)^. A placement for the inhibition of CYP3A4 was constructed at Rings A-D(C/K)-E(J)-F-I-H-R (Fig. 6G). Sitting of the ethyl part at Rings C and D was, however, unstable to support the trigger action. Lack of the intense inhibition of talarozole was consistent with the experimental result in recombinant CYP3A4 system^45^^)^.

A benzothiazole ring of talarozole was expected to undergo CYP3A4-mediated oxidation. A placement for the ring oxidation was available at Rings A(B)-D-K-Q-W’(U/T)-S (Fig. 6H). Another placement flipped the benzothiazole ring was also constructed (Data not shown).

In addition, a placement for the terminal-site oxidation of the ethyl part of talarozole was generated at Rings A-D(C)-E-K-QW’U(T) (Fig. 6I). The ethyl group located at rear-side would be oxidized to yield the primary alcohol, if the triazole ring served for triggering. Thus, talarozole containing a triazole part did not inhibit CYP3A4 intensely, but was suggested to undergo CYP3A4-mediated oxidations.

### 3.5 Placements of Imidazole Fungicides

Similar to triazole fungicides, chemicals containing imidazole inhibit catalytic activities of CYP forms through the interactions of the nitrogen lone pair with their heme parts. Triazole fungicides are resistant to CYP-mediated oxidation of the 1,2,4-triazole ring, while imidazole-containing chemicals are often metabolized by CYP forms to yield the *C*-2, *C*-4 and/or *C*-5 oxidized metabolites.

**Fig. 7.**
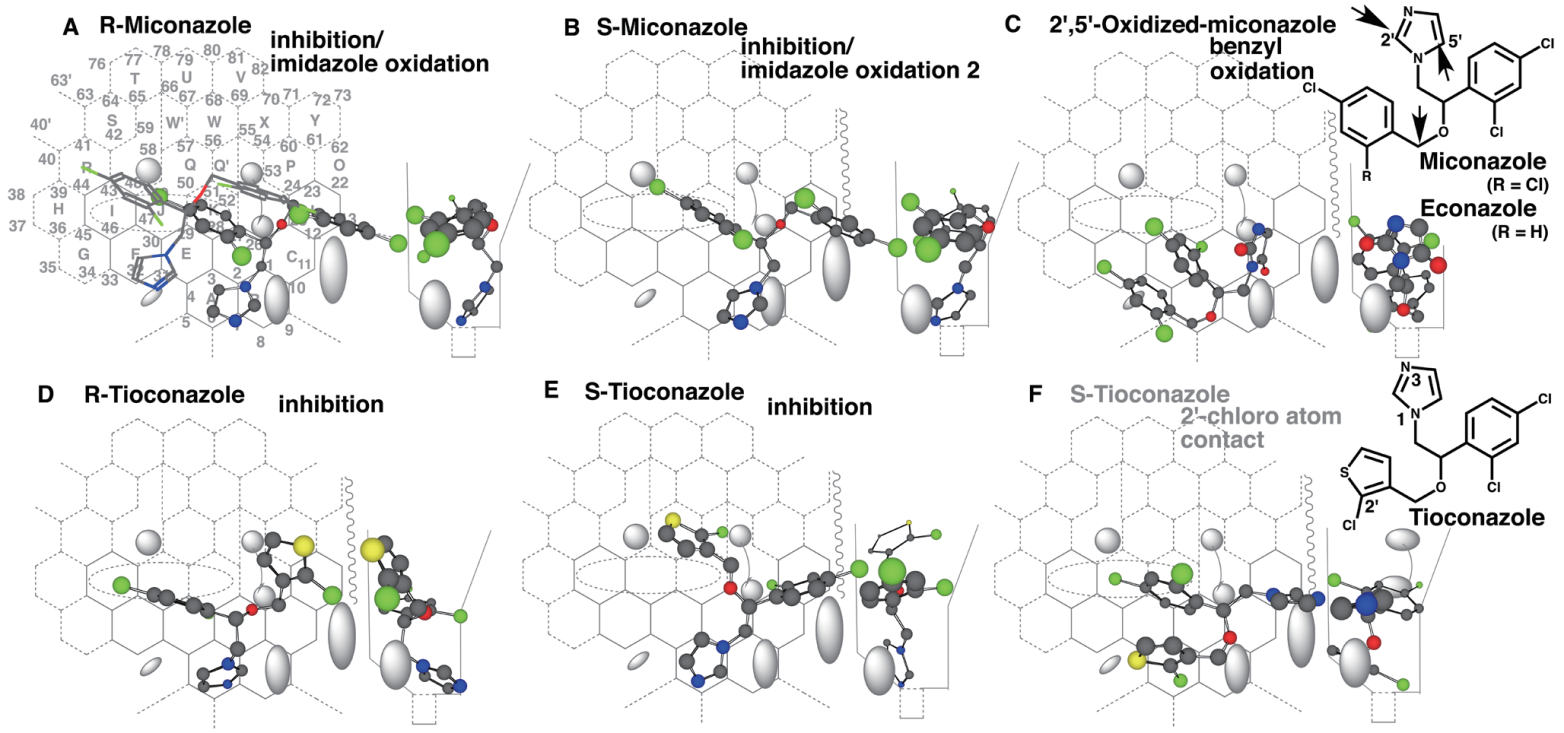
Placements of typical imidazole fungicide Placements for the inhibition and oxidation of imidazole ring of R- (A) and S-miconazole (B) and for the benzyl oxidation of 2’,5’-oxidized miconazole (C) are shown as cylindrical-shapes of 3D-structures. Placements for the inhibition of R- (D) and S-tioconazole (E) and for a sitting of 2’-chloro part of S-tioconazole at Site of oxidation are also shown on Template. Although not shown as a figure, the sitting of 2’-chloro part of R-tioconazole at Site of oxidation is also constructed.

Econazole (Fig. 7) undergoes the imidazole oxidations into the 2,4-dioxo-, 2-hydroxy-5-oxo- and 2,5-dioxo derivatives in humans and excreted in the urine as glucuronides after the *O*-dealkylations^46^^)^.

Instead of 4-chlorobenzyloxy part in econazole, miconazole contains 2,4-dichlorobenzyloxy part in the molecule. Miconazole is also metabolized through the imidazole oxidations in rats^47^^)^. The higher plasma disappearance of the S(+)-form than R(-)-form of miconazole was observed in rats^48^^)^. Placements for the inhibition of R- and S-miconazoles were available at Rings A-B-C(L-M)-D-K-J (Fig. 7A) and at Rings A-B-C(L-M)-D-K-J (Fig. 7B) Both molecules were able to pass the gate between Bay-1 and Cavity-2 residues. Both the placements might also contribute to the imidazole-ring oxidation due to the lack of futile sitting^18^^)^. Debenzylated metabolites of imidazole ring-oxidized miconazole is detected *in vivo*^49^^)^. A placement for the debenzylation was constructed at Rings A(B-D)-E-J plus Bay-1 (Fig. 7C), suggesting the possible role of CYP3A4 on the debenzylation.

Tioconazole containing 2-chlorothienyl group is also used as racemate^50^^)^. A placement of the inhibition of R-tioconazole was available at Rings A-D(C-L-P)-E-J-I plus Cavity-2. The 2-chlorothienyl group sat at facial-side without interfering the descending of Cavity-2 residue. Inhibition of S-tioconazole was generated at Rings A-D(C-L-M)-K-Q-Q’ (Fig. 7E). The 2,4-dichlorophenyl group of R-tioconazole managed to pass the gate between Bay-1 and Cavity-2 residues, although not expected to be efficient. Tioconazole is known to resist oxidations of the imidazole ring^51^^)^. The interactions with the 3-nitrogen atoms of the imidazole part with heme ceased the rotations for the inhibitions (Fig. 7D and E). The sittings of R- and S-tioconazole for the *C*-oxidations after the flipping of the imidazole ring, however, suggested the Futile-sitting from the localization at Rings A. The difference in ways of sittings from miconazole’s imidazole (non-rotatable, Fig. 7A and B) would affect the lack of the imidazole oxidation of tioconazole.

A placement for the oxidation of the 2-chlorothienyl part was constructed at Rings A-B-C(L-M)-D-K-J (Fig. 7F). The thienyl ring moved up, if the methylene oxy part was forced to shift left to avoid the collision with Front-residue. Even if the triggering would be achieved, the chlorine atom sat at Site of oxidation was thus unlikely to be oxidized. Consistent with these results, tioconazole is excreted by the non-oxidation pathway, *N*-glucuronidation, in humans^51^^)^.

## 4 Discussion

**Supplement Fig. 1:**
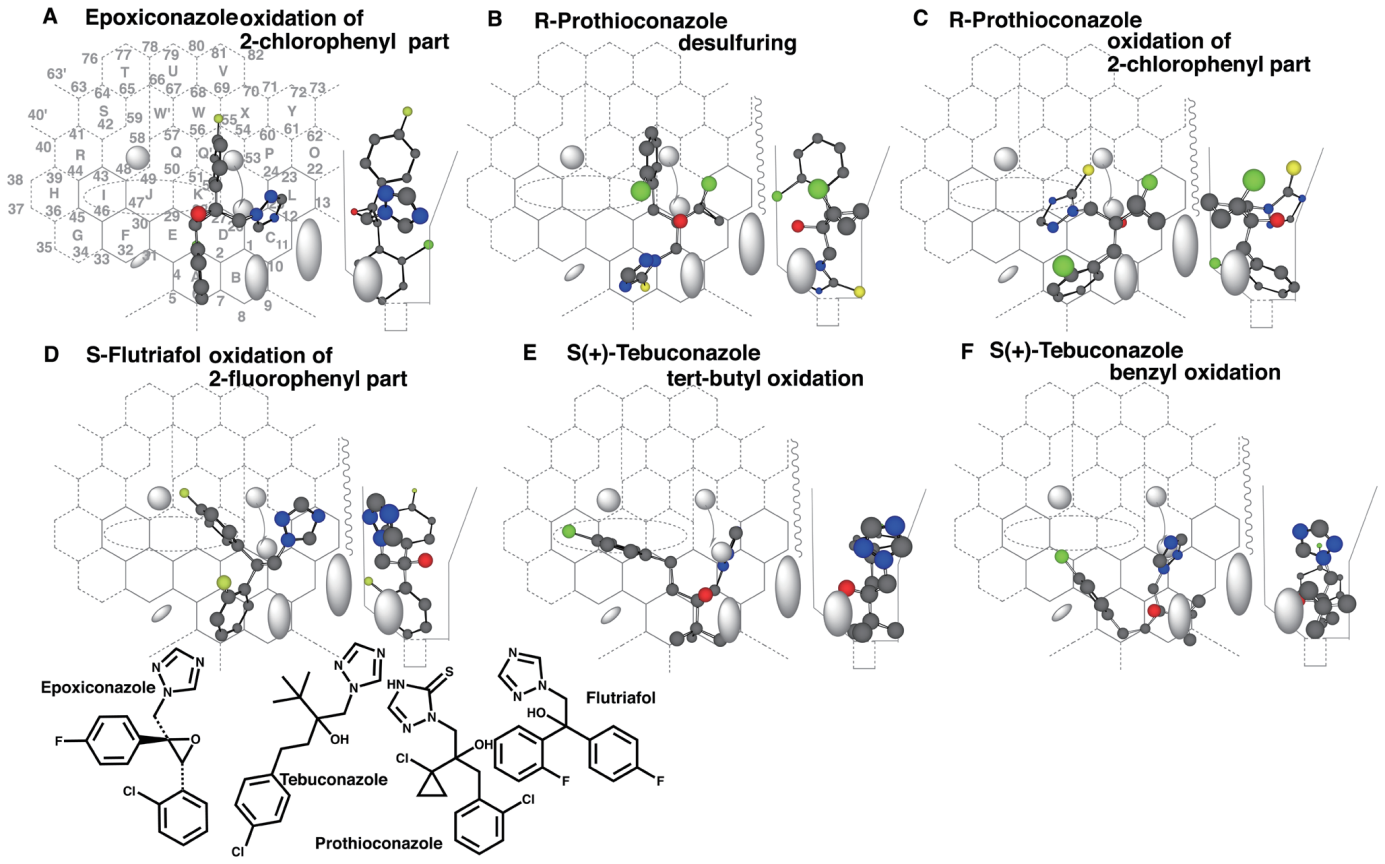
CYP3A4 is likely to participate in metabolisms of ortho-substituted aromatic rings ofazoles. Placements for the oxidation of 2-chlorophenyl part of epoxiconazole (A), for thedesulfuring (B) and oxidation of 2-chlorophenyl part (C) of R-prothioconazole and forthe oxidation of 2-fluorophenyl part of S-flutriafol (D) are available on Template.Placements of Stebuconazole for oxidations of the tert-butyl (E) and chlorobenzyl parts(F) are generated on Template. Preferential oxidations of the S-form than the R-form areexpected from the triazole sitting on Template.

More than twenty azole fungicides have been applied on CYP3A4 Template in the present study. Parts of Template-application data are shown in **Supplement **Figs. 1 and2. Clotrimazole and ketoconazole were already examined as inhibitors of CYP3A4 in our previous study^18^^)^. Interactions with Site of oxidation (Position of heme-access) of their triazole or imidazole parts were reconstituted on Template for all the azole stereoisomers tested, except for talarozole (Fig. 6). The placement of talarozole suggested the unstable sitting of the flexible 2’-ethylbutyl part at Trigger-site (Position 26). These results are consistent with a general concept that azole nitrogen-atoms interact with CYP3A4, but also show that the interaction at Rings I/J/K and Trigger site are also necessary for their intense inhibitory actions.

Data on the oxidation of azole fungicides in humans are often not available, and the data accessible are limited on experimental animals. In the present study, results of azole fungicides on Template were mostly consistent with the *in vivo* data on experimental animals, particularly on rats. These results suggest the major role of CYP3A forms on their oxidative metabolisms of azole fungicides in both humans and rats, although other CYP forms such as CYP2C9 and CYP2C19 also contribute^35^^)^. Slight differences in substrate specificities are also known between human CYP3A4 and rat CYP3A1/2^35^^,^^52^^)^. Careful experiments are thus necessary for the reliable prediction.

Azole fungicides are mostly used as enantiomers or diastereomers, which are probably due to the cost-effectiveness. These stereoisomers sometimes showed clear differences in their CYP3A4-mediated metabolisms. On CYP3A4 Template system, propiconazole ω-oxidation was expected to occur most efficiently on the 2S,4S-form (Fig. 4). The ω-1 and ω-2 oxidations would be proceeded preferably on the 2S,4R- and 2R,4R-forms, respectively. The oxidation leading to the dioxolane ring-cleavage was expected to be most efficient with the 2S,4R-form. These phenomena might underlie the profile of regioselectivity of CYP3A4-mediated oxidation of azole fungicides like propiconazole. CYP3A4 mediates oxidations of S-, but not R-myclobutanil^34^^)^, although CYP3A4 is inhibited by both the isomers (Fig. 3). CYP3A4 is expressed in small intestines and livers of humans. After a trace amount of exposure of myclobutanil-racemate from foods, intestinal CYP3A4 metabolizes preferentially S-myclobutanil and thus chances to be taken into systemic circulation may be higher for R-myclobutanil. These results suggest the relative higher persistency of R-myclobutanil than of the S-form in the body.

Free triazole is known as a metabolite of triazole fungicides containing a methylene bridge/spacer between triazole and a rest of the fungicide-molecule in experimental animals and humans. The triazole excretion is most prominent in rats and the higher amounts are detected in the male than the female. These results suggest the role of male-dominant enzyme^53^^,^^54^^)^ on the release of triazole from the fungicide.

An inverse relationship was observed on relative amounts of free triazole in excreta and relative contributions of CYP3A-mediated side-chain oxidations on total metabolisms of the triazole fungicides in rats (Table 1). Free triazole was detected clearly on fungicides having only few metabolic pathways like simeconazole and tetraconazole, and low levels of triazole were detected even with fungicides having multiple routes of side-chain oxidations. On the metabolism of these fungicides, at least one of the stereoisomers was judged refractory to CYP3A4-mediated oxidations. In addition, placements for the oxidation of the methylene bridge part were available for all the azoles refractory to the side-chain oxidations on Template (typical examples are shown as Figs. 3H, 5G, and5I). On CYP3A4-mediated metabolisms of steroids and PAHs^14^^,^^15^^)^, placement-types on Template are associated with observed abundance-orders of the metabolites in recombinant CYP3A4 systems. As described above, extents of oxidations of the aliphatic (other than azole and halophenyl) parts tended to correlate inversely with the triazole-release on metabolisms of triazole fungicides in rats (Table 1). The placement for triazole-release is thus not a favored one among the available, and possible to compete with the placement for inhibition. These factors may contribute to difficulties of the triazole-release detection in recombinant CYP3A4 systems. These observations prompt us to note the possible involvement of CYP3A forms on the triazole-release in experimental animals and humans. Of course, further studies are necessary to verify the possibility.

**Table 1.** A possible link of triazole-release with CYP3A function

Name	CH_2_ bridge	Triazole/counter parts	References
Type-1			
Cyproconazole	Yes	++	^55^
Difenoconazole	Yes	+	^56^
Fenbuconazole	Yes	+	^57^
Fluconazole*	Yes	+	^26^
Flusilazole	Yes	+++	^40^
Flutriafol	Yes	++	^58^
Genaconazole*	Yes	No data available	^59^^,^^60^
Hexaconazole	Yes	+	^61^
Ipconazole	Yes	No data available	^62^
Metconazole	Yes	+	^39^
Myclobutanil	Yes	No counter-part is detected	^32^
Penconazole	Yes	++	^63^
Propiconazole	Yes	+	^38^
Simeconazole	Yes	+++	^64^
Tebuconazole	Yes	+	^65^
Tetraconazole	Yes	+++	^66^
Voriconazole	Yes	++	^31^
Prothioconazole**	Yes	+	^67^
Type-2			
Bitertanol	No	Not detected	^68^
Neticonazole	No	Not detected	^69^
Paclobutrazol	No	Not detected	^70^
Triadimefon	No	Not detected	^42^
Type-3***			
Epoxiconazole	Yes	No data available	^71^
Diniconazole	No	++ (F > M)	^72^
Uniconazole	No	++	^43^

Excretions of triazole or the counter parts are less than 10% (+), 10 to 50% (++) and more than 50% of the total metabolism (+++).

*excreted mainly in urine as unchanged.

**The release of triazole is expected after the initial desulfuring.

***Type-3 triazoles are unable to take placements for triazole-release due to the difficulties to take bended structures. Rather higher amounts of triazole excretion was detected in female rats than the male with diniconazole. These observations suggest the distinct mechanism of the triazole-release for Type-3 triazole fungicides.

In addition, only the role of CYP3A4 was studied on azole fungicides in the present study. We already established Template systems of seven human CYP forms, and are currently studying other CYP forms such as CYP2C9 and CYP2C19. The relative contribution of different CYP forms may become predictable after the establishments of these Template systems.

## Template Terms Used in This Study

**2D and 3D:** two-dimensional and three-dimensional

**Adaptation:** Movements from the initial placement to stable placement by ways of irregular sittings; for example, movement left to right of ligands around Rings A and B

**Bay 1 and Bay 2:** CYP3A4 residue located lower left and right of Template

**Bi-molecule and Uni-molecule binding:** Interactions on Template with single molecule and with Trigger- and Pro-metabolized molecules

**Cavity 1 and Cavity 2:** Holes in the middle of Template. The residues in the holes are expected to participate in the IJK-Interaction and triggering.

**Front Residue:** Protein residue existing from facial side at Ring B, controlling the slide down phenomena to Site of Oxidation of the substrate

**Functional and non-functional placements:** Ligand placements leading to metabolite productions or inhibitions (functional), and withdrawing the activity through interactions with CYP residues (non-functional)

**Futile-sitting:** A phenomenon associated with lack of oxidations of rotatable and non-substituted phenyl group of ligands

**Groove:** A space for ligand sittings located beneath of Width-gauge

**IJK-Interaction:** Interaction of ligands with Rings I, J or K region, expected to initiate Forward-movement of ligand

**Right-side movement:** Right-side shift of ligands entered in Rings A and B to Bay-2 direction.

**Site of Oxidation:** A confined space of enzymatic catalysis. An area near Position 6 corresponds to Site of Oxidation in CYP3A4 Template.

**Slide-down:** Downward movement of the molecule to sit at Site of Oxidation

**Trigger molecule:** A molecule, which is not oxidized but acts for triggering the catalysis. Trigger molecules need to have direct contacts to pro-metabolized molecules on 2D Template.

**Trigger-site:** Position 26 of Template, which works to hold Cavity 2 residue. The interaction serves to initiate the catalysis.

**(Upper)-Cavity-2-residue:** A CYP3A4 residue sitting in Cavity-2 around Position 53, which is expected to go down to Position 26 for triggering.

**Width-gauge:** A guide shape for maximal width of ligand space Overlapping of ligands on line of Width-gauge is accepted.

## Supplement

**Supplement Fig. 2:**
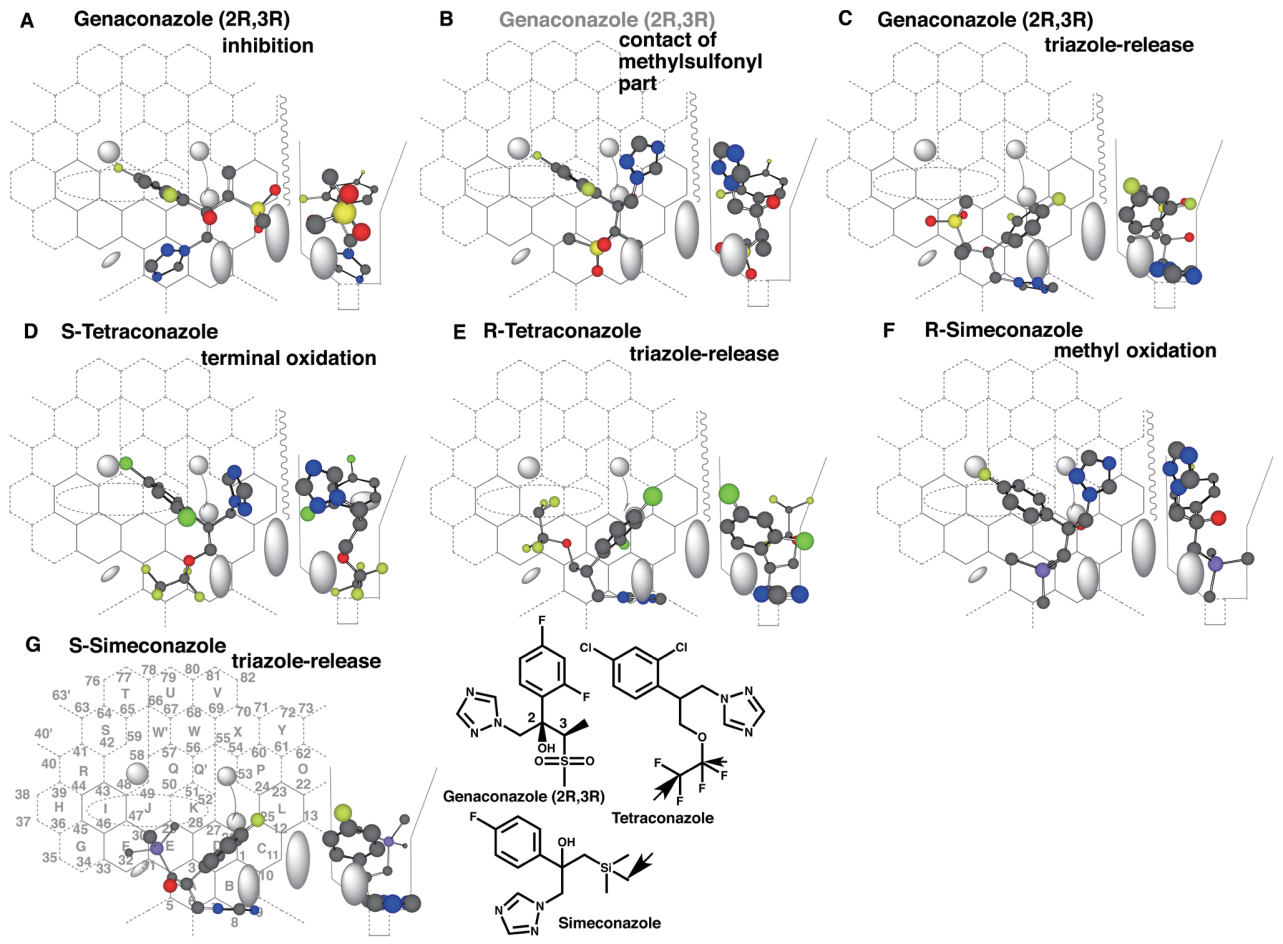
Interactions of CYP3A4 with 2R,3R-genaconazole, tetraconazole and simeconazole. Placements of 2R,3R-genaconazole for the inhibition (A), interaction at themethylsulfonyl part (B) and possible triazole-release (C) are constructed on Template.No metabolite production is expected from the interaction at the methylsulfonyl part.Placements of Stetraconazole for the terminal-oxidation (D) and of R-tetraconazole forthe triazole-release are generated. Placements for the methyl oxidation ofR-simeconazole (F) and for the triazole-release of S-simeconazole (G) are alshown onTemplate. The low efficiencies of CYP3A4-mediated oxidations of the side-chains oftetraconazole and simeconazole are likely to draw out the less preferred-order of theplacement for triazole-release.
